# Atypical Posterior Myocardial Infarction With Minimal Symptoms: Diagnostic Impact of Point-of-Care Ultrasound

**DOI:** 10.7759/cureus.107446

**Published:** 2026-04-21

**Authors:** Waleed Khalid Khalafallah Khalid, Saad Tariq Aziz, Hind Abdelazim Mirghani Ibrahim, Hamid Zafar

**Affiliations:** 1 Emergency Medicine, Queen Elizabeth Hospital, London, GBR; 2 Emergency Medicine, Royal College of Emergency Medicine, London, GBR; 3 Emergency Medicine, Ahfad University for Women, Khartoum, SDN

**Keywords:** acute coronary syndrome, atypical myocardial infarction, emergency ultrasound, missed myocardial infarction, point of care ultrasound, posterior myocardial infarction, regional wall motion abnormality, subtle ecg changes

## Abstract

Posterior myocardial infarction (PMI) is frequently under-recognised due to its non-classical electrocardiographic (ECG) presentation and variable symptom profile. We report a case of a 39-year-old male patient presenting with minimal chest pain and subtle ECG changes, in whom bedside point-of-care ultrasound (POCUS) played a pivotal role in diagnosis. Despite an initial low-risk clinical impression and non-diagnostic ECG findings, POCUS revealed regional wall motion abnormalities, prompting urgent cardiology reassessment. Subsequent transthoracic echocardiography confirmed inferior wall hypokinesia, and coronary angiography identified a proximal right coronary artery lesion due to plaque erosion, requiring percutaneous coronary intervention. This case underscores the importance of maintaining a high index of suspicion in atypical presentations and highlights the value of POCUS in early diagnosis, risk stratification, and timely clinical decision-making.

## Introduction

Posterior myocardial infarction (MI) is a recognised but frequently underdiagnosed subtype of acute coronary syndrome due to its indirect electrocardiographic (ECG) presentation. Unlike classical ST-segment elevation MI, posterior MI often manifests as ST-segment depression in anterior leads, which may be misinterpreted as non-ST-segment elevation MI (NSTEMI) [1,2].

Traditional ECG teaching characterises posterior MI by ST-segment depression in leads V1-V3 (typically ≥2 mm), accompanied by dominant R waves (R/S ratio > 1), representing the reciprocal changes of posterior ST-segment elevation and Q waves, respectively. T waves are classically upright (positively deflected) [3,4].

Contemporary literature, consistent with European Society of Cardiology recommendations, supports more sensitive diagnostic thresholds, whereby isolated ST-segment depression ≥ 0.5 mm in leads V1-V3 may warrant further evaluation for posterior MI, without the need for the full classical ECG pattern. Nevertheless, clinical practice often remains influenced by traditional teaching, with clinicians expecting the complete set of textbook features before considering posterior involvement. Consequently, posterior leads (V7-V9) are underutilised unless these classical changes are present, potentially leading to missed or delayed diagnosis in patients with subtle ECG findings. Delayed diagnosis may result in missed opportunities for timely reperfusion and increase the risk of adverse outcomes, including arrhythmias and impaired myocardial function [1].

We present a case of posterior MI in a patient with no apparent cardiovascular risk factors, minimal symptoms, and subtle ECG changes that did not fulfil classical teaching criteria. In this setting, point-of-care ultrasound (POCUS) proved decisive, prompting early diagnostic consideration and reinforcing clinical suspicion. This led to expedited referral and transfer to a cardiac centre, where coronary angiography ultimately confirmed the diagnosis, highlighting the critical role of POCUS in detecting atypical presentations.

POCUS has become an increasingly valuable tool in emergency medicine, enabling trained clinicians to perform focused bedside cardiac assessment in real time. In patients presenting with chest pain, POCUS can provide immediate adjunctive information by identifying regional wall motion abnormalities, thereby supporting clinical and ECG suspicion of acute coronary syndrome. In addition, it can assist in the rapid evaluation of alternative life-threatening diagnoses, such as aortic dissection, through visualisation of an intimal flap or assessment of aortic root dilatation. The availability of bedside ultrasound allows timely decision-making, particularly in settings where immediate formal echocardiography or specialist input may not be readily accessible.

## Case presentation

A 39-year-old male patient with no significant past medical history and not on regular medications presented to the emergency department with intermittent chest discomfort over four days. The pain was initially severe (7/10), waking him from sleep at approximately 1:00 AM on the day of presentation, and was associated with diaphoresis and nausea, with no vomiting. By the time of arrival at the emergency department at 4:00 AM, the pain had significantly improved, with a reported intensity of 1/10.

He was physically active, regularly attending the gym, with a body mass index (BMI) of 22 kg/m² and no evidence of central obesity. He was a light smoker (<10 cigarettes/day) and non-diabetic and had no history of hypertension, chronic kidney disease, or atrial fibrillation. There was no personal or family history of premature cardiovascular disease (defined as angina or MI in a first-degree relative under 60 years). He also had no history of autoimmune disease (including rheumatoid arthritis or systemic lupus erythematosus), migraine, or mental illness and was not taking any medications, including corticosteroids, antipsychotics, or phosphodiesterase-5 inhibitors. Despite a low baseline cardiovascular risk profile, the patient’s presentation corresponded to an estimated HEART score of 6, indicating a moderate risk in the acute setting [5].

On examination, he was haemodynamically stable: temperature 36.8°C, heart rate 86 bpm, blood pressure 144/80 mmHg, respiratory rate 18 breaths/min, and oxygen saturation 98% on room air. Cardiovascular and respiratory examinations were unremarkable.

**Figure 1:**
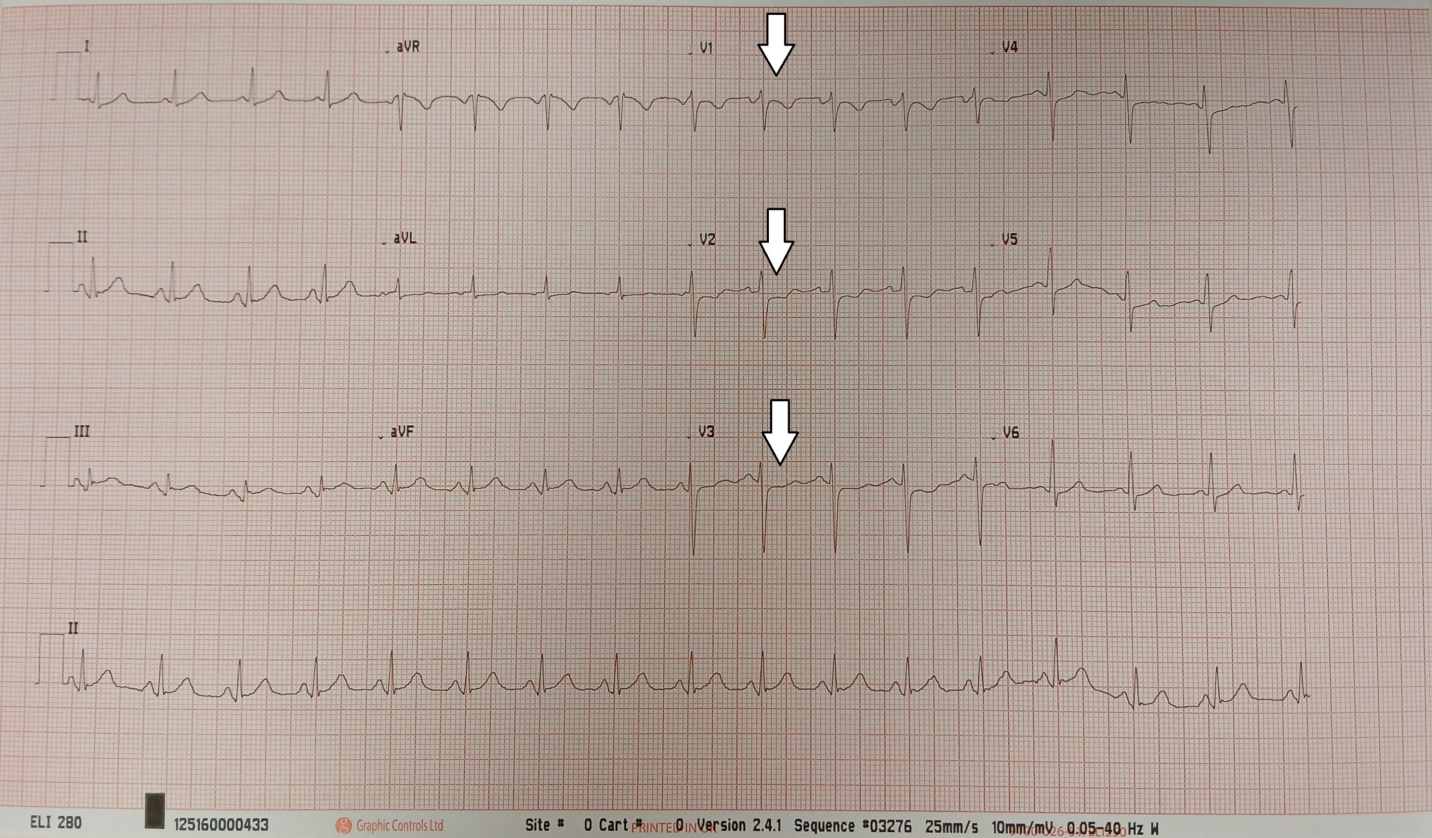
Initial 12-lead electrocardiogram Twelve-lead electrocardiogram demonstrating approximately 1 mm ST-segment depression in leads V1-V3 (arrows), with relatively tall but non-dominant R waves (R/S ratio < 1), not fulfilling classical criteria for posterior myocardial infarction.

Initial ECG demonstrated approximately 1 mm ST-segment depression in leads V1-V3 with associated tall R waves; however, the R/S ratio was <1, and T waves in V1 were not upright. While these findings were concerning for posterior myocardial ischaemia, they did not clearly fulfil classical criteria mandating immediate reperfusion, leading to initial uncertainty regarding urgent invasive management versus conservative NSTEMI treatment (Figure 1).

**Figure 2:**
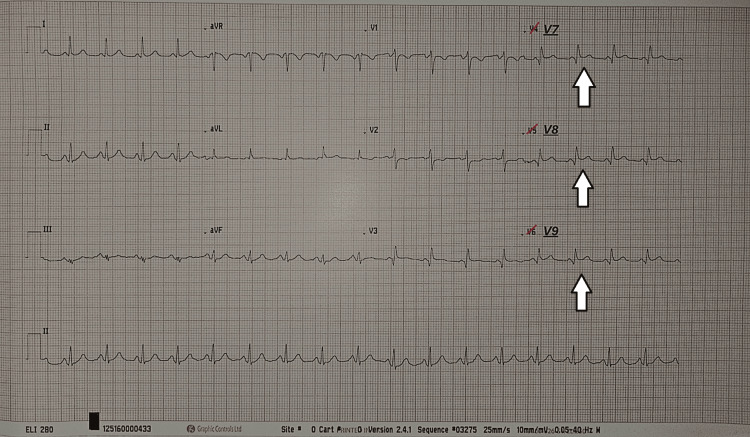
Posterior electrocardiogram (leads V7-V9) Posterior leads demonstrating ST-segment elevation in V7-V9 (arrows), supporting the diagnosis of posterior myocardial infarction.

Posterior leads (V7-V9) demonstrated ST elevation of approximately 1 mm in V7 and V8, and 0.5 mm in V9, supporting the diagnosis (Figure 2).

Initial high-sensitivity troponin was markedly elevated (>1,000 ng/L; upper reference limit 14 ng/L). The patient was treated according to local acute coronary syndrome protocol with aspirin and ticagrelor.

Despite these findings, and given the relatively mild symptoms and non-classical ECG features, discussion with the on-call cardiology team concluded that the ECG did not fulfil traditional criteria for posterior MI (in particular, ST depression < 2 mm, absence of upright T waves in V1, and R/S ratio < 1). The patient was therefore initially considered lower risk and managed conservatively with local admission as NSTEMI, rather than immediate transfer for invasive management.

**Figure 3:**
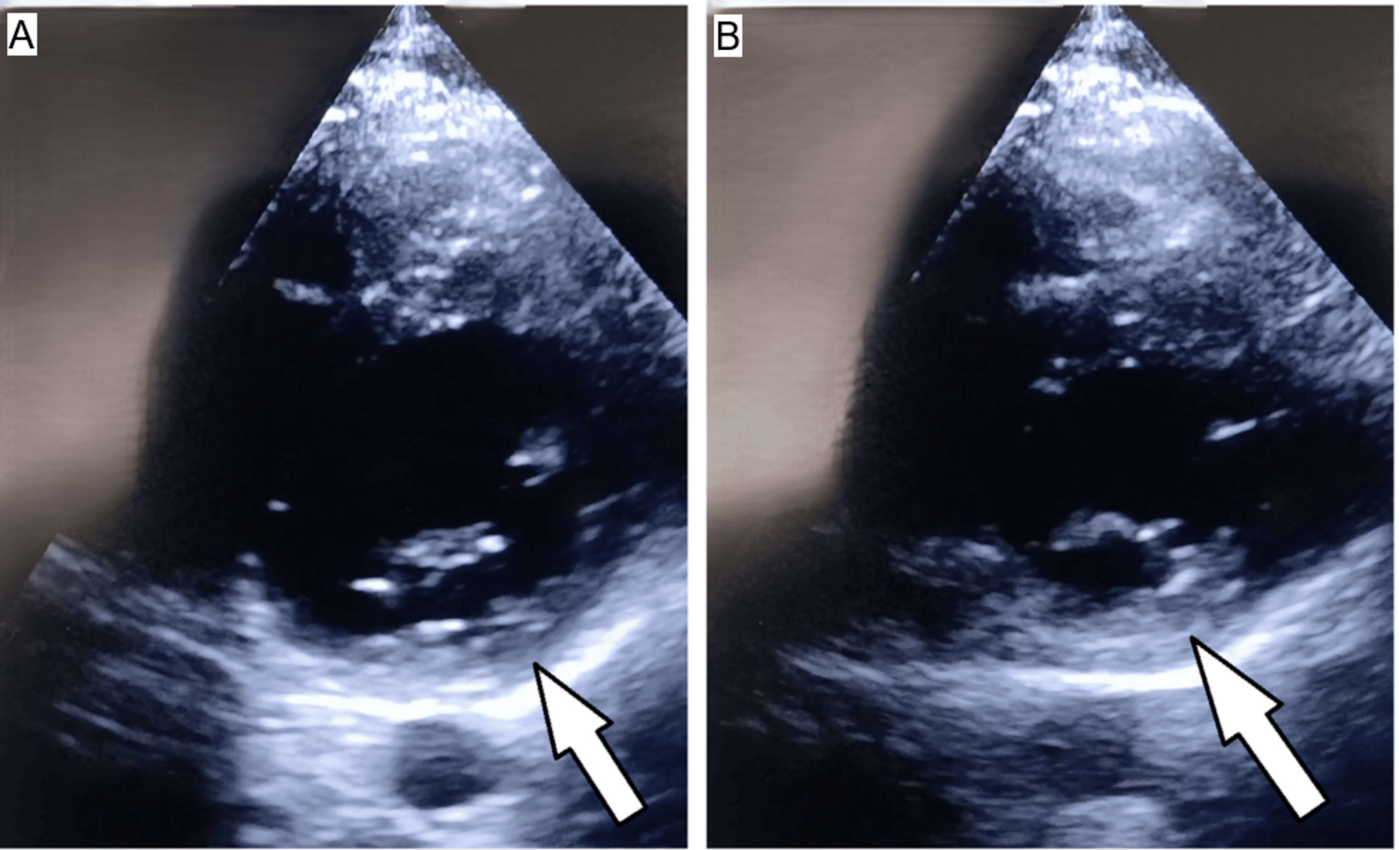
(A, B) Bedside point-of-care echocardiography (parasternal short-axis view) End-diastolic and end-systolic frames demonstrating reduced systolic thickening and inward motion of the inferior/inferolateral wall (arrows), corresponding to the posterior myocardial territory on electrocardiography (ECG), consistent with regional wall motion abnormality (hypokinesia).

However, due to ongoing clinical concern and the discrepancy between the markedly elevated troponin and the ECG appearance, bedside POCUS was performed in the emergency department using a phased-array transducer with a standard cardiac preset (1-5 MHz). This demonstrated clear regional wall motion abnormalities involving the inferior/inferolateral wall, corresponding to the posterior myocardial territory on ECG (Figure 3).

In light of these findings, the cardiology team was re-contacted, and the presence of regional wall motion abnormalities prompted escalation of care. The patient was subsequently accepted for urgent transfer to a tertiary cardiac centre with a working diagnosis of posterior MI. The ambulance arrived within approximately 10 minutes, and the patient was transferred promptly to the cardiac centre.

Formal transthoracic echocardiography performed at the cardiac centre confirmed hypokinesia of the basal to mid inferior and inferolateral walls. Coronary angiography demonstrated a dominant right coronary artery with a proximal lesion showing moderate (50%-74%) stenosis. Optical coherence tomography (OCT) identified features consistent with plaque erosion. The patient underwent successful percutaneous coronary intervention with deployment of a 3.5 × 28 mm drug-eluting stent, achieving final TIMI 3 flow with good stent expansion and apposition. The patient remained clinically stable post-procedure and was discharged after two days with appropriate secondary prevention, smoking cessation advice, and referral to cardiac rehabilitation.

## Discussion

Posterior MI remains a diagnostic challenge due to its indirect ECG presentation. Classical ECG findings include ST-segment depression in leads V1-V3 with dominant R waves, representing a mirror image of posterior ST elevation [3,4].

Guideline-based criteria, including those from the European Society of Cardiology, suggest that ST depression ≥ 0.5 mm in V1-V3, particularly when accompanied by ST elevation ≥ 0.5 mm in posterior leads (V7-V9), is sufficient to diagnose posterior MI [1,2]. However, these findings are often subtle, and posterior leads are not routinely recorded, contributing to under-recognition [5].

Despite this, clinical practice often remains influenced by traditional teaching [5], where greater ST depression (e.g., ≥2 mm), dominant R waves (R/S ratio > 1), and upright T waves in V1 are expected before considering posterior MI. Such expectations are not required by current guidelines and may lead to missed or delayed diagnosis in patients with less pronounced ECG changes [3,6].

In addition, clinical judgement may be influenced by patient characteristics. Younger patients with minimal cardiovascular risk factors are often perceived as lower risk, which may reduce clinical suspicion for MI and favour conservative management strategies, such as treatment for NSTEMI with local admission rather than early invasive evaluation.

This case highlights the limitations of relying solely on ECG findings, symptom severity, and perceived risk profile. The patient presented with minimal symptoms, subtle ECG changes, and low calculated cardiovascular risk, yet was found to have an acute coronary occlusion requiring revascularisation. It also highlights the gap between traditional ECG teaching and contemporary guideline-based interpretation, where posterior MI may be under-recognised despite suggestive findings.

POCUS played a pivotal role in this case [7]. The identification of regional wall motion abnormalities provided direct, real-time evidence of myocardial ischaemia, which proved more compelling in clinical decision-making and facilitated re-discussion with the cardiology team, ultimately leading to urgent transfer and definitive management [8]. One important limitation of POCUS is that regional wall motion abnormalities may persist after a prior MI, which reduces its specificity in distinguishing acute from chronic findings. This highlights the importance of interpreting POCUS findings within the full clinical context. In this context, plaque erosion is increasingly recognised as a distinct mechanism from plaque rupture and is more commonly observed in younger patients and smokers presenting with acute coronary syndromes.

In many centres, formal echocardiography is performed after admission and may be subject to delays. In contrast, bedside echocardiography performed in the emergency department allows immediate assessment, particularly in diagnostically uncertain or “grey-area” cases, thereby expediting decision-making and potentially reducing time to reperfusion [8]. Current guidelines recommend formal transthoracic echocardiography primarily for the assessment of complications of acute MI, such as left ventricular dysfunction or mechanical complications. However, they do not define a specific role for POCUS in the early emergency department assessment or decision-making pathway of acute coronary syndromes. In addition, ECG interpretation in acute MI can be challenging, particularly when attempting to localise the infarct-related territory [9].

The use of POCUS in this context may have important clinical implications. Early identification of myocardial ischaemia can prevent delays in definitive management and reduce the risk of adverse outcomes, including malignant arrhythmias, cardiac arrest, or the development of heart failure due to delayed revascularisation [1]. This can be explained by the ischaemic cascade, in which abnormalities in myocardial contractility occur before ECG changes. As a result, regional wall motion abnormalities detected by POCUS may be evident before classical ECG findings become apparent [10].

This case also highlights that ECG changes in MI may evolve over time and may not always be apparent in the early stages [11]. Spontaneous reperfusion may lead to transient symptom improvement, while the underlying unstable plaque remains at risk of re-occlusion and thrombus propagation.

Emergency clinicians should therefore maintain a high index of suspicion and avoid over-reliance on classical ECG criteria or perceived low-risk profiles. Adjunctive tools such as POCUS can play a crucial role in identifying high-risk pathology in atypical presentations and should be increasingly integrated into emergency medicine training and practice.

## Conclusions

Posterior MI may present with minimal symptoms and subtle ECG changes, leading to under-recognition, particularly when traditional criteria are applied. Posterior leads and POCUS improve diagnostic accuracy, with early detection of regional wall motion abnormalities facilitating timely escalation and reperfusion. Consideration of posterior lead recording in patients with anterior ST-segment depression may further enhance diagnostic accuracy. However, POCUS remains operator-dependent and requires adequate training and experience.

## References

[1] Ibanez B, James S, Agewall S (2018). 2017 ESC guidelines for the management of acute myocardial infarction in patients presenting with ST-segment elevation: the Task Force for the management of acute myocardial infarction in patients presenting with ST-segment elevation of the European Society of Cardiology (ESC). Eur Heart J.

[2] O'Gara PT, Kushner FG, Ascheim DD (2013). 2013 ACCF/AHA guideline for the management of ST-elevation myocardial infarction: a report of the American College of Cardiology Foundation/American Heart Association Task Force on Practice Guidelines. Circulation.

[3] Levis JT (2015). ECG diagnosis: isolated posterior wall myocardial infarction. Perm J.

[4] Wung SF, Drew BJ (2001). New electrocardiographic criteria for posterior wall acute myocardial ischemia validated by a percutaneous transluminal coronary angioplasty model of acute myocardial infarction. Am J Cardiol.

[5] Six AJ, Backus BE, Kelder JC (2008). Chest pain in the emergency room: value of the HEART score. Neth Heart J.

[6] White LD, Wall J, Melhuish TM, Vlok R, Lee A (2017). Recognition and management of posterior myocardial infarction: a retrospective cohort study. Br J Cardiol.

[7] Labovitz AJ, Noble VE, Bierig M (2010). Focused cardiac ultrasound in the emergent setting: a consensus statement of the American Society of Echocardiography and American College of Emergency Physicians. J Am Soc Echocardiogr.

[8] Mahmoud MZ (2017). Echocardiography in the evaluation of chest pain in the emergency department. Pol J Radiol.

[9] Kontos MC, Desai PV, Jesse RL, Ornato JP (1997). Usefulness of the admission electrocardiogram for identifying the infarct-related artery in inferior wall acute myocardial infarction. Am J Cardiol.

[10] Nesto RW, Kowalchuk GJ (1987). The ischemic cascade: temporal sequence of hemodynamic, electrocardiographic and symptomatic expressions of ischemia. Am J Cardiol.

[11] Nable JV, Brady W (2009). The evolution of electrocardiographic changes in ST-segment elevation myocardial infarction. Am J Emerg Med.

